# A Primary Extracutaneous Presentation of Merkel Cell Carcinoma

**DOI:** 10.7759/cureus.28088

**Published:** 2022-08-16

**Authors:** Hussain Dalal, Ayesha Iqbal, Pooja Rani, Jamal Saaed, Dmitriy Berenzon

**Affiliations:** 1 Internal Medicine, Nuvance Health, Poughkeepsie, USA; 2 Internal Medicine, Vassar Brothers Medical Center, Poughkeepsie, USA; 3 Hematology and Medical Oncology, Vassar Brothers Medical Center, Poughkeepsie, USA

**Keywords:** merkel cell carcinoma, immunohistochemistry, hepatic mass, pleural effusion, stage iv disease

## Abstract

Merkel cell carcinoma (MCC) is a rare aggressive cutaneous neuroendocrine malignancy with a mortality rate of around 33%. The presence of advanced disease at the time of diagnosis is associated with poor prognosis. Twofold etiologies have been described in the pathogenesis of Merkel cell carcinoma: chronic exposure to ultraviolet (UV) light and Merkel cell polyomavirus (MCPvY). MCC usually affects sun-exposed skin areas, and the presence of cutaneous nodules is the hallmark of the disease. However, there have been case reports in the literature where the diagnosis of MCC was made in the absence of any cutaneous findings. We present a case report of Merkel cell carcinoma that is unique in its presentation because of the presence of pulmonary and hepatic nodules and the absence of cutaneous lesions.

## Introduction

Merkel cell carcinoma (MCC) is an aggressive neuroendocrine malignancy of the skin that predominantly affects the elderly population [1]. The sun-exposed areas of the body are usually affected, suggesting ultraviolet (UV) light-mediated pathogenesis. However, another virus-mediated pathogenesis has been associated with Merkel cell carcinoma, and that is Merkel cell polyomavirus (MCPvY). MCPvY is the only polyomavirus associated with human cancer and is usually associated with 80% of MCC in immunocompromised hosts [2]. The two etiologies share common clinical, histopathological, and prognostic characteristics [3]. MCC usually presents as solitary cutaneous or subcutaneous nodules in the sun-exposed areas. UV exposure has been postulated to be causing DNA damage in both viral-mediated and nonviral-mediated carcinogenesis [3]. Although cutaneous findings are a hallmark of MCC, there have been a handful of cases reported in the literature about MCC with no cutaneous findings. We describe one such rare case with a diagnosis of MCC in a patient who presented with hepatic and pulmonary nodules without cutaneous involvement.

## Case presentation

**Figure 1:**
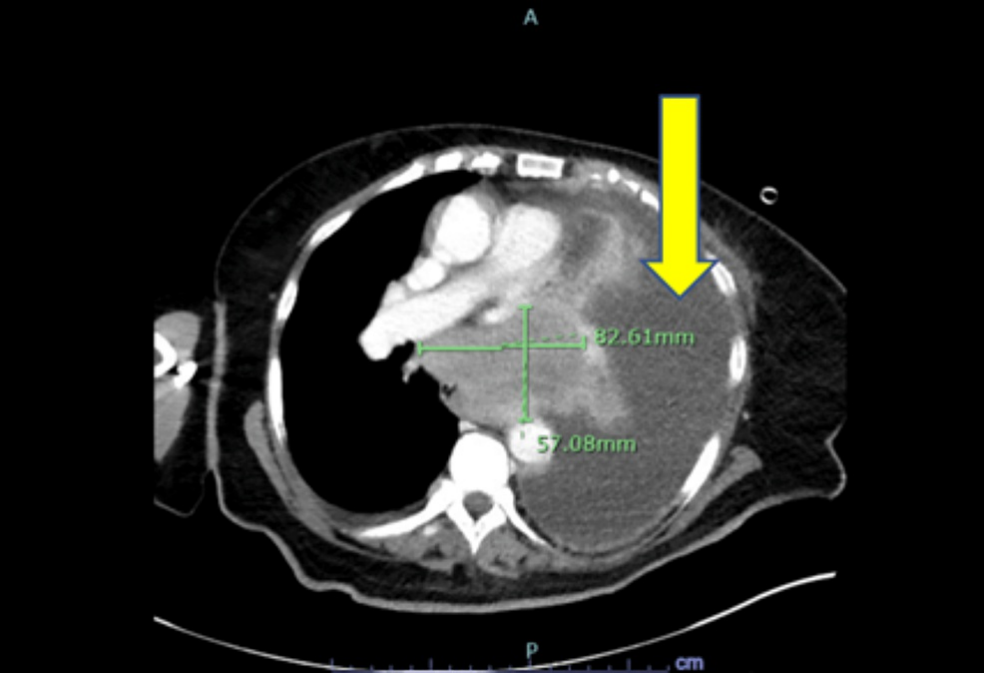
Chest CT showing huge mediastinal mass (measurements as noted) along with massive left-sided pleural effusion (arrow)

**Figure 2:**
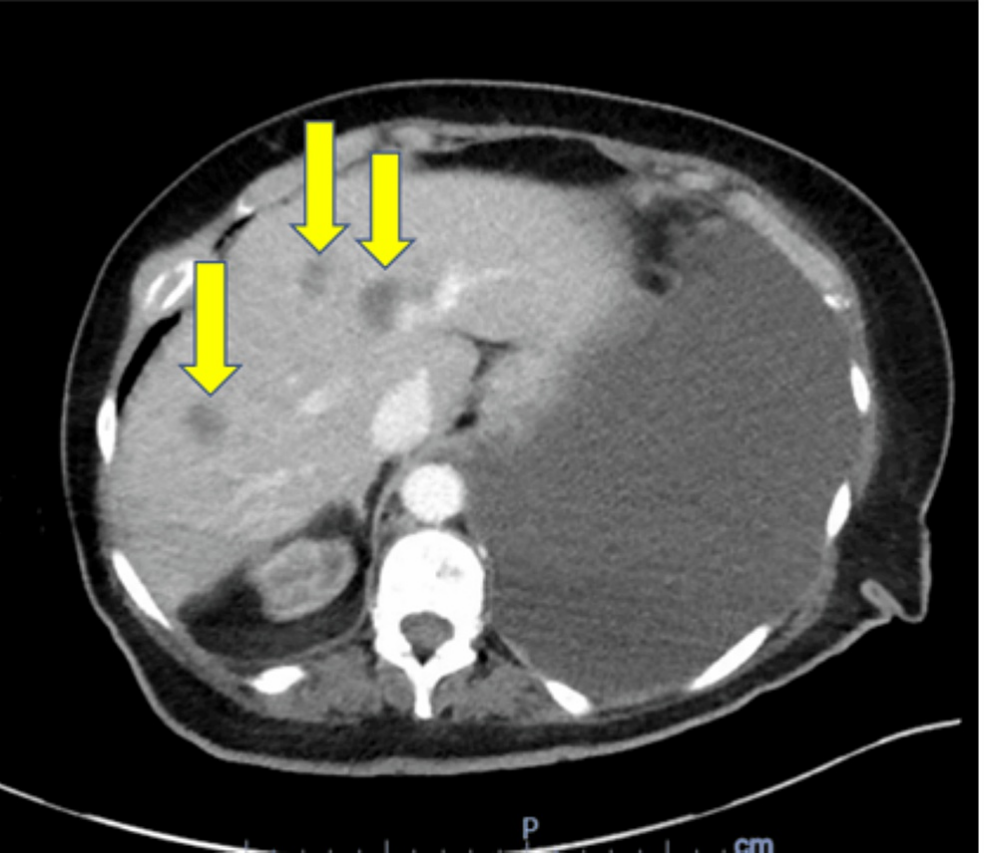
Abdominal CT showing multiple metastatic lesions (arrows)

**Figure 3:**
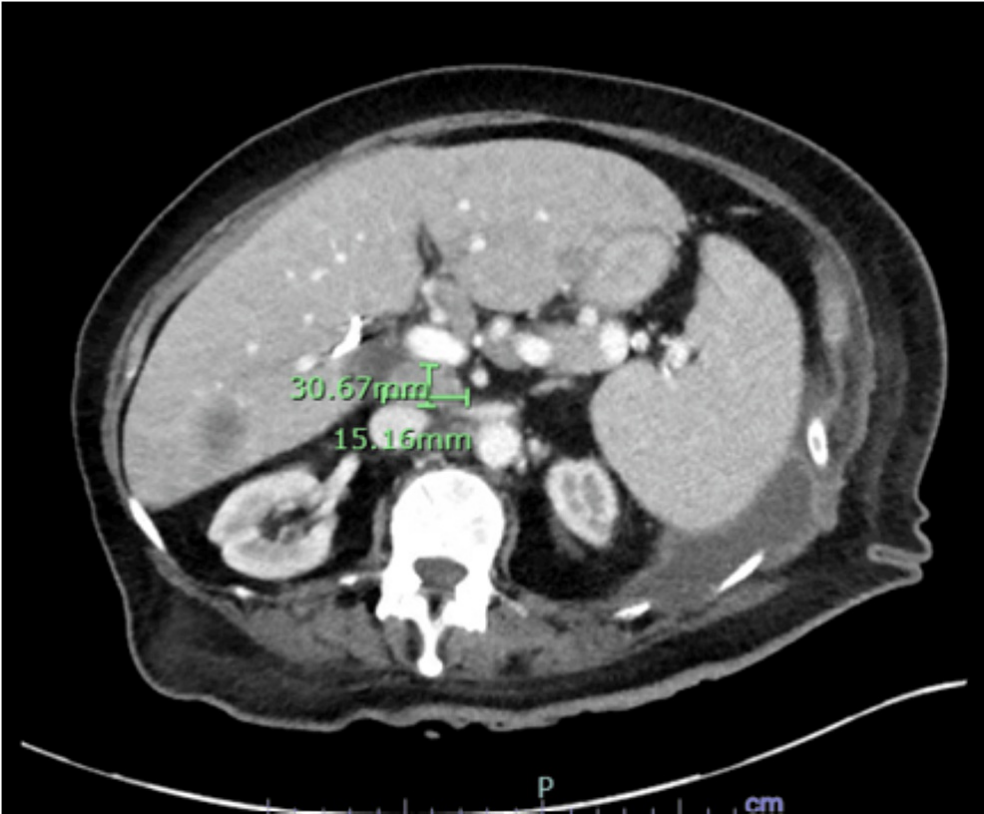
Abdominal CT showing portacaval and aortocaval lymph nodes

**Figure 4:**
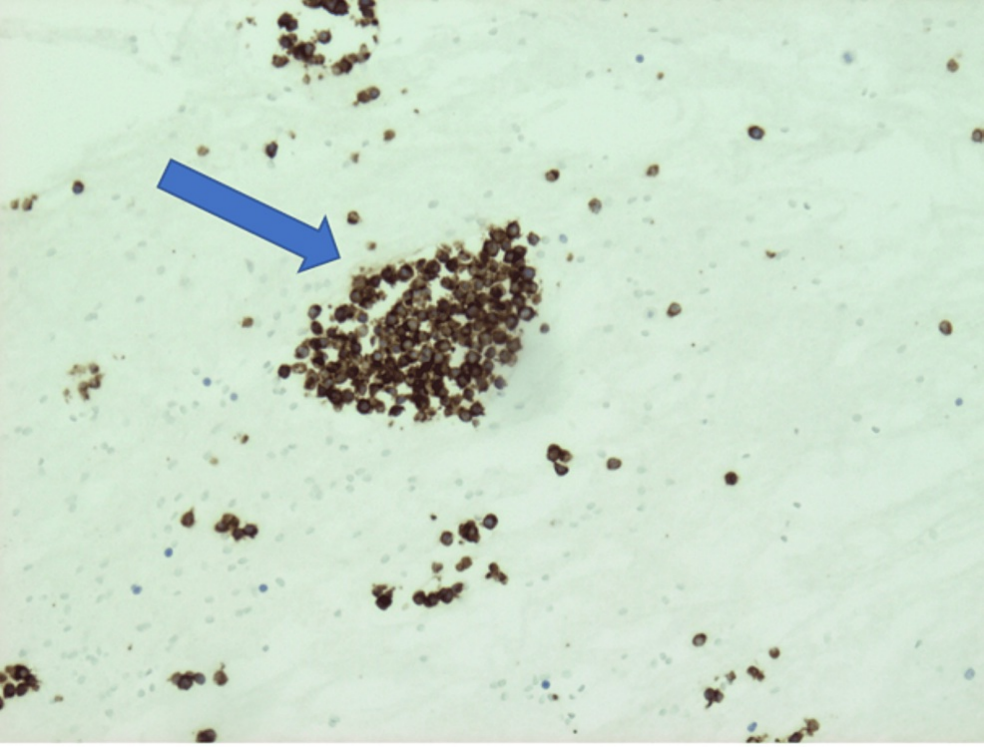
Synaptophysin staining of Merkel cell carcinoma on pleural fluid cytology (arrow)

**Figure 5:**
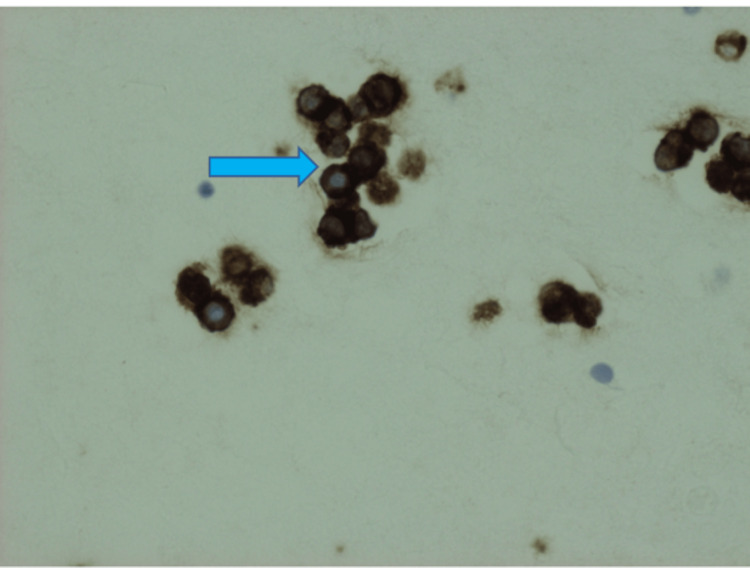
CK20 staining cytology of pleural fluid (arrow pointing toward Merkel cell carcinoma cells staining positive for CK20)

**Figure 6:**
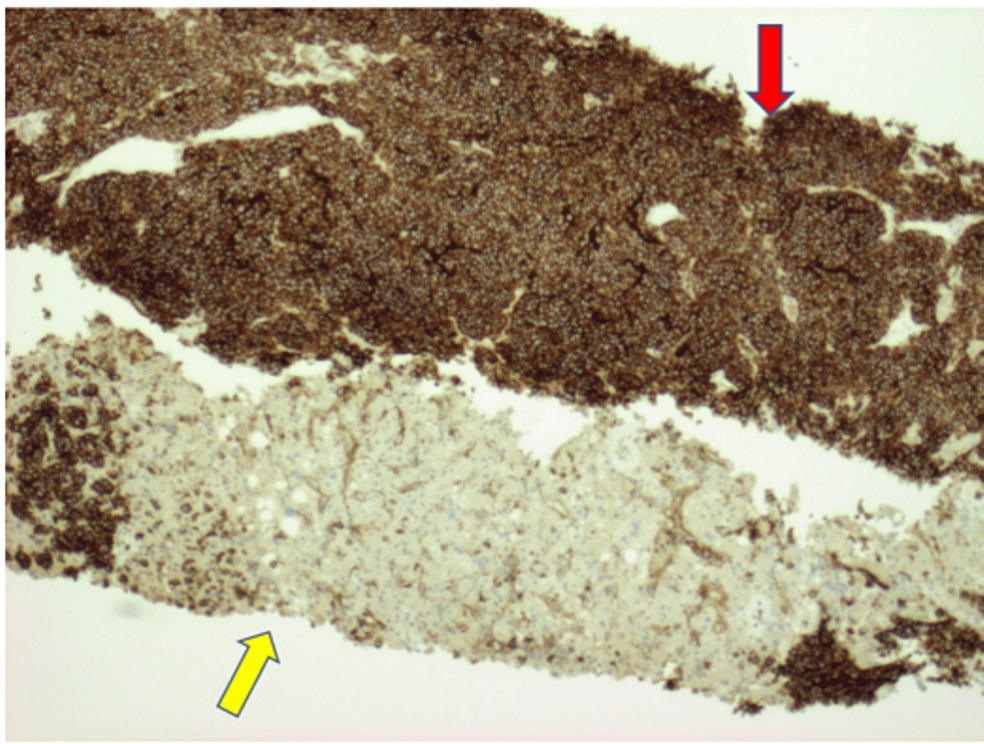
CD56 staining of the liver mass showing Merkel cell carcinoma stain (red arrow) adjacent to normal liver parenchyma (yellow arrow)

**Figure 7:**
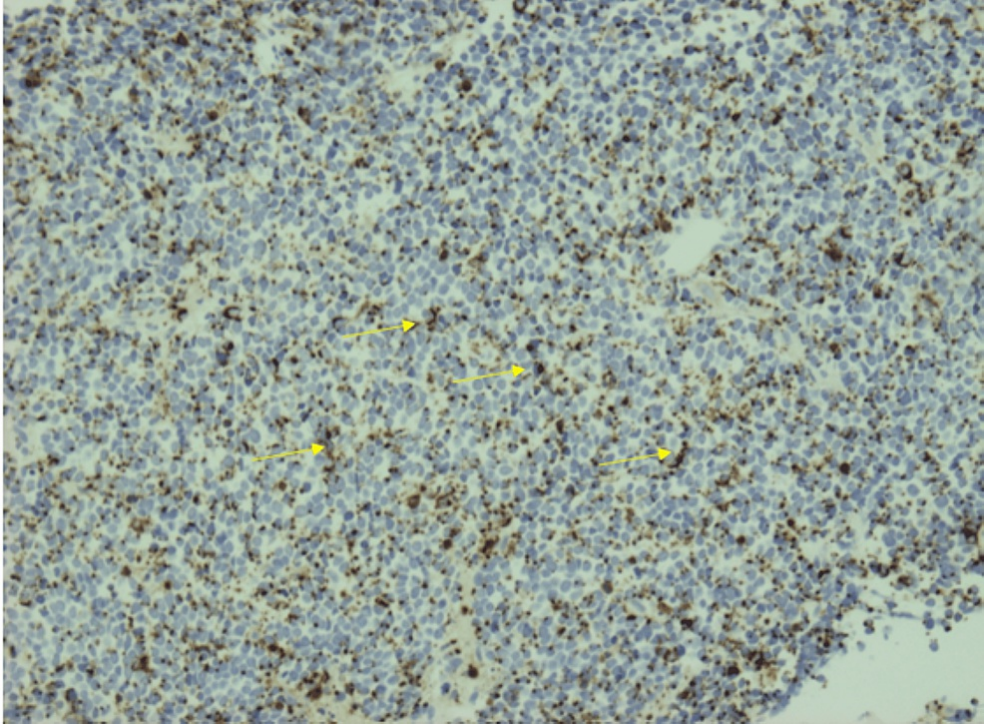
Staining with CK20 with the classic “dot-like” pattern for the liver mass (arrow)

Our patient is an 83-year-old female, ex-smoker, with a medical history of hypertension, hyperlipidemia, and type II diabetes who presented to the emergency department with a one-week history of shortness of breath and orthopnea. One week prior to presentation, she was treated for community-acquired pneumonia with antibiotics at another facility and was subsequently discharged. However, due to further worsening shortness of breath, she was sent to our institution for evaluation. The patient reported weight loss and decreased appetite over the last few months, but no associated symptoms of night sweats or chills were reported. On presentation, she was afebrile and hemodynamically stable but hypoxic with oxygen saturations in the high 80s requiring supplemental oxygen. Chest X-ray was remarkable for left pleural effusion; subsequently, chest/abdomen/pelvis CTA revealed a large hilar mass along with left pleural effusion, pleural nodularity, liver hypodensities, and surrounding lymphadenopathy (Figures 1-3). The patient underwent ultrasound-guided thoracentesis with the removal of 2 L strawberry to cranberry color fluid. Pleural fluid analysis was positive for RBCs and elevated protein. After thoracocentesis, she continued to be hypoxic, and a repeat chest X-ray showed reaccumulating pleural fluid. Hence, she underwent IR-guided pleural catheter placement. A liver biopsy was performed for a definitive diagnosis. Pleural fluid pathology came back positive for epithelial neoplasm with neuroendocrine features suspicious for Merkel cell carcinoma. The immunohistochemical stains performed on the cell block section were positive for CAM5.2, cytokeratin (CK) AE1/3, cytokeratin 20 (Figure 4), synaptophysin (Figure 5), and CD56 (Figure 6) in tumor cells and negative for cytokeratin 7, thyroid transcription factor-1 (TTF-1), CD45, and chromogranin and show an estimated Ki-67 proliferative index of 20%. The right lobe liver mass biopsy was positive for neuroendocrine cells as well, favoring Merkel cell carcinoma. Immunohistochemical stains were positive for cytokeratin AE1/3 and cytokeratin 20 (Figure 7), with a Ki-67 proliferative index of 70%-80% in tumor cells.

Due to the highly aggressive nature of the disease and the patient’s extremely poor performance status, she was not deemed to be a candidate for chemotherapy or immunotherapy. After an extensive conversation with the patient and her family, it was decided to focus on comfort care. The patient was transitioned to inpatient hospice and passed two days later.

## Discussion

Merkel cell carcinoma was first described as a carcinoma of the skin exhibiting carcinoid features in 1972 by Cyril Toker [2]. On pathological review of MCC, similarities to Merkel cells of the skin were noted, which contain neurosecretory granules and are involved in light touch stimuli [2,4]. In 2013, MCC had an incidence rate of 0.7 cases per 100,000 persons in the United States, corresponding to roughly 2,500 cases [5]. The incidence increases exponentially with age, with approximately 10 per 100,000 person-years in individuals aged 85 years or older [5]. Between 2000 and 2013, there has been a substantial increase in MCC cases compared to melanoma and other solid tumors [5].

The pathogenesis of MCC is not very well understood. Merkel cells are found in the epidermal layers, and they play a role in tactile sensation; however, they do release certain hormones, and their hormonal function remains unclear. MCC usually affects the dermis, sparing the epidermis, and the appearance of “small round cells” in the dermal layers makes MCC difficult to distinguish from other malignancies histologically [6]. Hence, immunohistochemical stains are quite useful in this case.

On immunohistochemistry, MCC shows positivity for low-molecular-weight keratin and neurofilaments, which appear as dot-like condensations of filaments. It is strongly positive for cytokeratin type 20 (CK20) with distinct dots concentrated around the nucleus and negative for thyroid transcription factor-1 (TTF-1), which helps designate MCC from histologically similar neoplasm such as small neuroendocrine tumors [7-9]. The demonstration of neuron-specific enolase and the absence of S100 protein leukocyte common antigen, vimentin, and HMB45 are confirmatory for the diagnosis of MCC [10,11]. In addition, MCC shows reactivity with chromogranin, synaptophysin, vasoactive intestinal peptide, substance P, pancreatic polypeptide, calcitonin, adrenocorticotropic hormone, somatostatin, other peptide hormones such as PAX-5, TdT, glypican-3, and CD117 [12-17]. Table 1 highlights the different stains used to distinguish MCC from other related malignancies. In our case, immunohistochemical analysis of the pleural fluid and liver biopsy was notably for “dot-like” cytokeratin AE1/3, cytokeratin 20 (Figures 6, 7), synaptophysin (Figure 4), and CD56 (Figure 5) but negative for chromogranin, cytokeratin 7, thyroid transcription factor-1, and leukocyte common antigen. Therefore, the neuroendocrine morphology was consistent with the diagnosis of MCC.

**Table 1:** Immunobiological markers of MCC

Marker	MCC	Lymphoma	Melanoma	SCLC
Cytokeratin 20 (CK20)	+	−	−	−
Cytokeratin 7 (CK7)	−	−	−	+
Chromogranin A	+/−	−	−	+/−
HBM45	−	−	+	−
Huntingtin-interacting protein-1 (HIP1)	+	+/−	−	−
Melan-A/MART-1	−	−	+	−
Leucocyte common antigen (LCA)	−	+	−	
S100B	−	−	+	−
Thyroid transcription factor-1 (TTF-1)	−	−	−	+
Neuron-specific enolase	+	−	−	+/−
Vimentin	−	+	+	−

As with any disease process, a good history and physical examination are paramount to the diagnosis. Our patient presented with acute hypoxic respiratory failure owing to the large unilateral pleural effusion and large mediastinal mass. Initial dermatologic examination, as well as follow-up dermatologic examination including the head and the face, did not reveal any suspicious lesions. The dermatologic assessment was done by the oncology team and multiple nurses during the shifts. A CT scan is one of the modalities that can be used for the evaluation of the MCC disease process [18]. For our patient, CT of the chest/abdomen/pelvis showed subcarinal mass extending into the left hilum occluding the left mainstem bronchus and multiple liver metastatic lesions. This scan eventually led to thoracocentesis and cytology of the pleural fluid, which was indicative of a neuroendocrine malignancy with a strong preference for MCC. The patient also underwent a biopsy of the liver mass, which was consistent with metastatic MCC. Clinically and histologically, the patient’s diagnosis of MCC was consistent with stage IV disease.

After an extensive literature review, we were able to find only a handful of articles that showed MCC with no cutaneous involvement. Siref et al. reported a case of MCC presenting as a visceral malignancy where there were no cutaneous findings [19]. Quiroz-Sandoval et al. reported a case of large retroperitoneal mass presenting as MCC, which was successfully excised [20]. As for our patient, she had neither personal nor family history of malignancy and presented with acute respiratory failure in the setting of pleural effusion with hilar mass and liver mass. This is a very unusual presentation of MCC with extracutaneous involvement, and to the best of our knowledge, no similar cases have been reported in the literature.

The diagnosis of MCC, especially at the advanced stage, carries a grim prognosis. Despite a much better understanding of the disease pathology, survival rates remain poor for advanced diseases [6]. While recent advances in immune checkpoint inhibitors and the availability of chemotherapy for stage IV disease have added new treatment options for MCC, our patient was a poor candidate for any form of intervention. From the time our patient presented, she was frail and had a very poor functional status. Given that she had evidence of liver metastasis, surgical intervention was not an option. After an extensive conversation with the patient’s family regarding options for chemotherapy/immunotherapy, the patient was transitioned to comfort care measures where she expired in a few days.

## Conclusions

Although considerable advancements in the disease pathogenesis and treatment options have been noted in the last few years for MCC, mortality continues to remain high, especially in advanced-stage disease. Our case highlights not only the poor prognosis of advanced-stage disease but also the unusual presentation of MCC. As mentioned earlier, only a small number of cases have been reported in the literature without cutaneous involvement. MCC presenting as a metastatic disease with mediastinal and hepatic involvement with extracutaneous findings is extremely rare. While there are a plethora of differential diagnoses of metastatic disease, our case highlights the importance of considering rare forms of malignancies such as MCC even when no cutaneous findings are noted.
